# Efficacy of single vs. multiple botulinum toxin type A injections for trigeminal neuralgia

**DOI:** 10.3389/fneur.2025.1570447

**Published:** 2025-08-26

**Authors:** Ying-ying Xu, Cheng-wei Guo, He-jing Chen, Ji-li Bao, Ying Li, Shu-yang Ma, Qi-lin Zhang, Jing Liu, Wei-feng Luo

**Affiliations:** Department of Neurology and Clinical Research Center of Neurological Disease, The Second Affiliated Hospital of Soochow University, Suzhou, China

**Keywords:** botulinum toxin type A, trigeminal neuralgia, repeated injection, multiple treatments, efficacy evaluation

## Abstract

**Background:**

The effectiveness of Botulinum toxin type A (BTX-A) has been established in trigeminal neuralgia (TN). This study aimed to assess the therapeutic efficacy and safety of BTX-A injection within a group of TN patients who have chosen to continue BTX-A therapy.

**Methods:**

This was a retrospective medical record-review study. Demographic and clinical features and severity and frequency of pain before and 4 weeks after the BTX-A administration were extracted from the patient files. BTX-A was injected into the painful area subcutaneously and/or submucosally. BTX-A injections were performed by the same physician using the same methods. Pain severity was assessed using the visual analog scale (VAS). The patient’s overall response to treatment was assessed using the Patient Global Impression of Change (PGIC). Patients were divided into two groups, single-treatment (TN-S) group and multiple-treatment (TN-M) group, according to the numbers of treatment.

**Results:**

Thirty patients were included in this study. We classified 16 (53.33%) as TN-S group and 14 (46.67%) as TN-M group. The median VAS score of all patients was 8 (6.75, 10) before the first treatment and 3 (2, 6.25) after the first treatment (*P* < 0.001). In the TN-M group, median of the difference of VAS before and after treatment of the first and the last treatment were 7 (5, 8) and 5 (2, 7.25), respectively, indicating a significantly better effect for the first treatment compared with the last treatment (*P* = 0.024). However, this difference in PGIC distributions between the two treatments was not significant (*P* = 0.070).

**Conclusion:**

In summary, BTX-A treatment was effective in TN. Elderly patients and patients with good response to the first treatment were more likely to choose to continue BTX-A multiple-treatments. BTX-A remains effective within a group of TN patients who have chosen to continue BTX-A therapy, but the efficacy decreases to a certain extent after multiple treatments.

## 1 Introduction

Trigeminal neuralgia (TN) is characterized by paroxysms of intense, stabbing pain in the distribution of the mandibular and maxillary divisions (rarely, the ophthalmic division) of the fifth cranial nerve, triggered by chewing, talking, or other activities (1, 2). TN is the most common facial pain neuralgia. On the basis of the 3rd edition of the International Classification of Headache Disorders (ICHD-3), TN is subclassified according to the presumed aetiology into idiopathic, classic, and secondary TN (3). Studies have shown that approximately 24%–49% of patients still experience persistent pain during the interictal period of painful attacks. The distribution area of such persistent pain coincides with that of paroxysmal pain, mostly presenting as burning sensation, pulsation, or dull pain. In the past, TN with this symptom was classified as type 2 or atypical. Now, it has been redefined as “TN with persistent pain” (1, 4). The condition affects more women than men, with a lifetime prevalence of 0.3% overall. The mean age of onset for TN is 52.9 years and the mean age of patients is 62.3 years (5).

First-line and second-line medical treatments for TN, which are typically high doses of antiepileptic drugs, often cause adverse events, such as sedation, fatigue, dizziness, coordination disturbances, and cognitive deficits (6). When pharmacological treatments are insufficient or accompanied by unacceptable side effects, surgical treatments can be considered. Surgical approaches used to relieve TN-related pain include microvascular decompression, percutaneous radiofrequency thermal rhizotomy, percutaneous ablation, and gamma knife radiosurgery (7, 8) However, surgical approaches are also associated with side effects, and new treatment regimens have emerged as surgical alternatives (8).

Since 2002, many studies have examined the use of botulinum toxin type A (BTX-A) as a safe and effective treatment for TN (9–13). In 2005, eight patients with TN were enrolled in an open-label study and 100 U of botulinum toxin was injected along the zygomatic arch region. All the patients expressed that there was a significant decrease both in the frequency and the severity of the pain approximately 3.2 ± 2 days after the injection (11). However, the deep position of the zygomatic region increases the difficulty of injection, and there is a risk of bleeding (11). Later, researchers preferred subcutaneous/submucosal or intradermal injections in the trigeminal nerve distribution area. In a randomized, double-blind, placebo-controlled study, 42 TN patients were randomly allocated into two group. The study showed that BTX-A significantly reduced the mean visual analog scale (VAS) scores at as early as week 2 compared to placebo. The effect was sustained throughout the course (12 weeks) of the study (12). Xiromerisiou et al. (13) assessed the effectiveness of a single course of BTX-A add-on to carbamazepine or oxcarbazepine in 15 treatment-refractory patients with TN. In all treated cases, a single BTX-A session proved effective and provided long-term effective relief.

However, the analgesic effects of BTX-A diminish over time (14), and patients may require repeated injection for continued pain management. Whether repeated BTX-A injections are associated with stable pain-reducing effects or more adverse reactions remains unknown. This study assessed the therapeutic efficacy and safety of BTX-A injection within a selected group of TN patients who have chosen to continue BTX-A therapy.

## 2 Materials and methods

### 2.1 Subjects

This was a retrospective medical record-review study. We retrospectively reviewed all files of patients diagnosed with TN referred to the Neurology Clinic and the Inpatient Department of the Second Affiliated Hospital of Soochow University for BTX-A administration between June 2019 and November 2021. The sample size of the study was based on available data and no statistical power calculation was conducted prior to the study. Patients with files that met the following criteria were included: (1) fulfilled the criteria of idiopathic or classic TN according to ICHD-3 (3), (2) had BTX-A administration after initial comprehensive clinical evaluation, (3) underwent a second comprehensive examination at the end of the 4th week after administration and had WeChat video follow-up visit every month, (4) had no prior exposure to BTX-A treatment, (5) the failure of conventional medical and surgical interventions (including microvascular decompression, percutaneous radiofrequency thermal rhizotomy, percutaneous ablation, and gamma knife radiosurgery) to manage pain (pain intensity mean scores ≥ 4) (6) excluded the patients with background interictal pain, (7) excluded any conditions that might potentially heighten the risks associated with BTX-A exposure (e.g., myasthenia gravis or motor neuron disease) or women who were pregnant, nursing, (8) had a stable dose of medication for TN during the entire study period, (9) excluded the patients who underwent surgical interventions after BTX-A treatment. Patients could receive BTX-A treatment once again if the VAS was greater than 4 and they were inclined to choose BTX-A treatment. A time interval of more than 3 months was necessary between the two treatment sessions. Patients were divided into two groups according to the numbers of treatment. Patients with single BTX-A treatment constituted TN with single-treatment (TN-S) group. Patients with two or more treatments were included in the TN with multiple-treatment (TN-M) group.

Study approval was obtained from the Clinical Research Ethics Committee of Second Affiliated Hospital of Soochow University (approval number JD-HG-2022-027). Written informed consent for BTX-A injections was obtained from all included patients before the treatment.

### 2.2 Assessments

The same physician, an expert in pain and BTX-A administration, evaluated all patients and completed patient files according to the same data sheet. These files contained information pertaining to medical history, clinical features associated with TN (i.e., etiology, onset, side, distribution, and previous treatments for TN), mood and the patient’s overall response before and 4 weeks after treatment, the volume of BTX-A administered. Pain severity was assessed using VAS. The patient’s overall response to treatment was assessed using the Patient Global Impression of Change (PGIC). PGIC was scored on a 7-point scale, including very much improved, much improved, minimally improved, no change, minimally worse, much worse, very much worse. Mood was assessed using the 14-item Hamilton Anxiety Scale (HAMA-14) and the 17-item Hamilton Depression Scale (HAMD-17). Patients were assessed all the scales at baseline and 4 weeks after treatment to obtain detailed information, which were parts of our standard care. During the following telephone visit, a 50% reduction in pain was defined as treatment effectiveness, and the duration of efficacy maintenance was recorded for each patient.

### 2.3 Interventions

BTX-A (100 U *Clostridium botulinum* type A neurotoxin complex, 5 mg gelatin, 25 mg dextran, and 25 mg saccharose) was commercially procured (Lanzhou Institute of Biological Products, Lanzhou, China) and diluted to 25 U/mL prior to administration. The BTX-A solution was injected in the facial pain area and the trigger point at a depth of 0.5 cm, 2.5–5 IU per point, separation of 15 mm and at 15–20 injection points, using 1-mL syringe. Given the risk of muscle weakness through diffusion, 5.0 units administered to the symptomatic side was injected in 2 points on the asymptomatic side to prevent asymmetry. At baseline, patients usually received medications (e.g., carbamazepine, gabapentin, or opioids) to alleviate their pain. These medications remained unchanged during the course of the study and no new treatment was given.

### 2.4 Statistical analysis

Shapiro–Wilk test was used to check whether the data conformed to the normal distribution before analyzing the data. The data conforming to the normal distribution were expressed as the mean ± standard deviation, and the independent sample *t*-test was used for comparison between the two groups. Data with skewed distribution were expressed as median (interquartile range), and the comparison between the two groups was performed using the Mann–Whitney U Test for two independent samples, and the comparison between the same sample and the same sample before and after treatment was performed using the nonparametric Test for two related samples (Wilcoxon signed-rank Test). Categorical variables were expressed as frequency (percentile), and chi-square test was used to compare the constituent ratios. All analyses were two-sided, and *P-*values of less than 0.05 were considered to indicate statistical significance. Statistical analysis was conducted with SPSS IBM Statistics, version 25.0 (SPSS Inc.).

## 3 Results

In total, 30 files were determined to fulfill the study criteria. The demographic and clinical features of the patients are summarized in Table 1. None of the patients had bilateral TN. The median age of TN-M was older than the median age of TN-S (*P* = 0.025). There was no significant difference in other clinical features between the two groups.

**Table 1:** Demographic and clinical features of patients.

Characteristic [*n* (%) or median (p25, p75)]	TN (*n* = 30)	TN-S (*n* = 16)	TN-M (*n* = 14)	*p-*value
Female, *n* (%)	16 (53.33%)	8 (50%)	8 (57.14%)	0.700
Age (years)	63 (57,76.25)	60.5 (56.25,70.5)	76.5 (59.25,80.5)	0.025
Duration of TN (years)	3 (1.38,5)	3 (1.25,7.25)	2.25 (1.38,4)	0.259
Systemic hypertension	14 (46.67%)	7 (43.75%)	7 (50%)	0.736
Side of TN, *n* (%)				0.518
Right	19 (63.33%)	11 (68.75%)	8 (57.14%)	
Left	11 (36.67%)	5 (31.25)	6 (42.86%)	
Branch site, *n* (%)				0.628
V1	3 (10%)	2 (12.5%)	1 (7.14%)	
V2	7 (23.33%)	4 (25%)	3 (21.43%)	
V3	8 (26.67%)	4 (25%)	4 (28.57%)	
V1 + V2	2 (6.67%)	2 (12.5%)	0	
V2 + V3	9 (30%)	4 (25%)	5 (35.72%)	
V1 + V2 + V3	1 (3.33%)	0	1 (7.14%)	
History of surgical interventions, *n* (%)	7 (23.33%)	4 (25%)	3 (21.43%)	0.821

*p*-value <0.05 was considered statistically significant. *P*: comparison between TN-S and TN-M.

TN, trigeminal neuralgia; TN-S, trigeminal neuralgia with single-treatment; TN-M, trigeminal neuralgia with multiple-treatment; Surgical interventions including microvascular decompression, percutaneous radiofrequency thermal rhizotomy, percutaneous ablation, and gamma knife radiosurgery.

The median VAS score of all patients was 8 (6.75, 10) before the first treatment and 3 (2, 6.25) after the first treatment (*P* < 0.001). The median pre-treatment VAS score of 9 (6.75, 10) for the TN-M patients was slightly higher than the median score of 8 (6.25, 10) for the TN-S patients (*P* = 0.423). After the first treatment, the median VAS score was 2 (1, 2.25) in the TN-M group and 6 (3.25, 8.75) in the TN-S group. In both groups, the reduction in the VAS score after treatment was statistically significant (*P* = 0.001 and *P* = 0.028, respectively).

The median of difference of VAS before and after treatment (D-value of VAS) was 7 (5, 8) in the first treatment of TN-M group, which was a significantly larger change than 0 (0, 3.5) in the TN-S group (*P* < 0.001). In the TN-S group, 6.25% of patients were “very much improved,” 12.5% were “much improved,” 18.75% were “minimally improved,” and 62.5% were “no change,” whereas, in the TN-M group, 35.71% of patients were “very much improved” and 64.29% were “much improved,” indicating that patients in the TN-M group experienced better outcomes in the first treatment than those in the TN-S group (*P* < 0.001) (Table 2). The HAMA and HAMD scores for the TN-S group were [4.5 (2.25, 7.75); 5 (3.25, 5.75)] before and [4.5 (2.25, 6); 5 (3.25, 5.75)] after the first treatment. The HAMA and HAMD scores for the TN-M group were [5 (2.75, 7); 4 (3, 7.75)] and [4.5 (2.25, 6.0); 5 (2.75, 6.5)]. The difference in HAMA and HAMD scores before and after the first treatment were not significant (all *P >* 0.05). The treatment efficacy duration for the TN-S group was 1.12 (0, 2.5) months, compared with the significantly longer treatment efficacy of 7.5 (6, 12) months for the first treatment in the TN-M group (*P* < 0.001) (Table 2).

**Table 2:** Assessments of BTX-A treatment in TN-S group and TN-M group.

Assessment [*n* (%) or median (p25, p75)]	TN-S (*n* = 16)	TN-M (first-treatment) (*n* = 14)	TN-M (last-treatment) (*n* = 14)	*P*_1_ -value	*P*_2_ -value
Dosage of BTX-A (U)	100 (85,100)	100 (80,100)	100 (80,100)	0.768	0.560
Pre-treatment VAS	8 (6.25,10)	9 (6.75,10)	8 (6,10)	0.423	0.216
Post-treatment VAS	6 (3.25,8.75)	2 (1,2.25)	2.5 (1.75,4.25)	< 0.001	0.109
D-value of VAS	0 (0,3.5)	7 (5,8)	5 (2,7.25)	< 0.001	0.024
Pre-treatment HAMA	4.5 (2.25,7.75)	5 (2.75,7)	6 (5,7)	0.690	0.655
Post-treatment HAMA	4 (2.25,6)	4.5 (2.25, 6.0)	6 (5,7)	0.529	0.349
Pre-treatment HAMD	5 (3.25,5.75)	4 (3,7.75)	6 (4.75,8)	0.719	0.450
Post-treatment HAMD	5 (4,5.75)	5 (2.75, 6.5)	7 (5.5,7.25)	0.525	0.430
Efficacy duration (months)	1.12 (0,2.5)	7.5 (6,12)	6 (3,7.75)	< 0.001	0.357
PGIC, *n* (%)				< 0.001	0.070
Very much improved	1 (6.25%)	5 (35.71%)	2 (14.29%)		
Much improved	2 (12.5%)	9 (64.29%)	9 (64.29%)		
Minimally improved	3 (18.75%)	0	1 (7.14%)		
No change	10 (62.5%)	0	2 (14.29%)		
Side effect	8 (57.14%)	7 (50%)	6 (42.86%)	0.351	0.432

*p*-value<0.05 was considered statistically significant.

*P*_1_: comparison between TN-S and the first-treatment of TN-M; *P*_2_: comparison between the first-treatment and the last-treatment of TN-M.

TN, trigeminal neuralgia; TN-S, trigeminal neuralgia with single-treatment; TN-M, trigeminal neuralgia with multiple-treatment; BTX-A, botulinum toxin type A; VAS, visual analog scale; HAMA, Hamilton Anxiety Scale; HAMD, Hamilton Depression Scale; D-value, the difference of VAS before and after treatment; PGIC, the patient global impression of change.

Systemic side effects were not observed after the first treatment for either group. Local swelling and muscle relaxation occurred in eight patients in the TN-S group and seven patients in the TN-M group, with no significant difference between groups. All side effects were mild and resolved within 2 months (Table 2).

**Figure 1:**
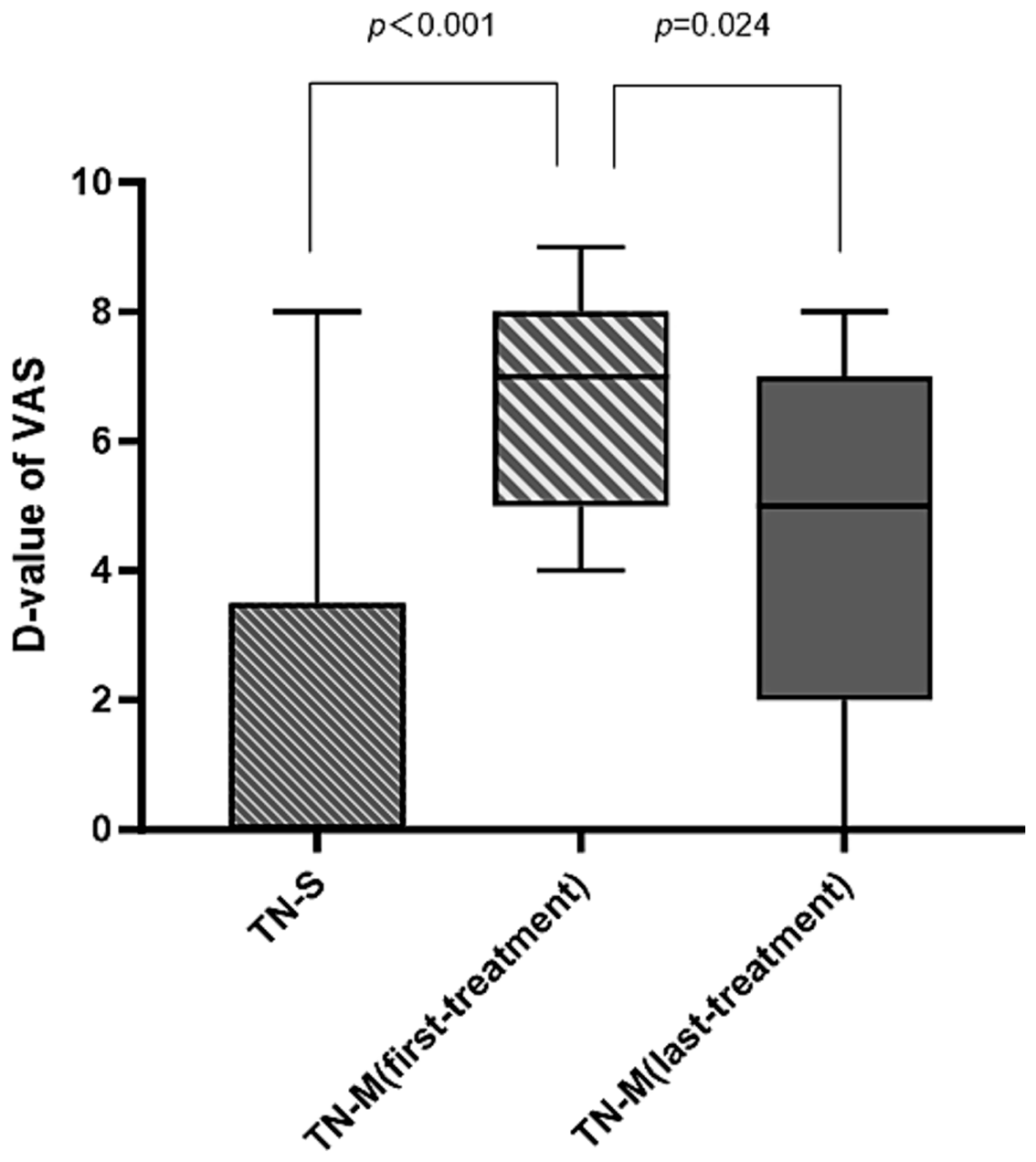
Comparative effectiveness of BTX-A treatment of TN-S and TN-M group. VAS, visual analog scale; D-value: the difference of VAS before and after treatment; BTX-A, botulinum toxin type A; TN-S, trigeminal neuralgia with single-treatment; TN-M, trigeminal neuralgia with multiple-treatment. *P*-value<0.05 was considered statistically significant.

In the TN-M group, median D-value of VAS of the first and the last treatment were 7 (5, 8) and 5 (2, 7.25), respectively, indicating a significantly better effect for the first treatment compared with the last treatment (*P* = 0.024) (Figure 1). The PGIC response after the first treatment revealed that 35.71% of patients were “very much improved” and 64.29% were “much improved,” whereas the results after the last treatment indicated that 14.29% of patients were “very much improved,” 64.29% were “much improved,” 7.14% were “minimally improved,” and 14.29% had “no change.” However, this difference in PGIC distributions between the two treatments was not significant (*P* = 0.070). The treatment efficacy duration for the first treatment was 7.5 (6, 12) months, which was longer than 6 (3, 7.75) of the last treatment with no significant difference (*P* = 0.357) (Table 2).

## 4 Discussion

The major findings from our study were as follows: (1) BTX-A treatment was effective in TN, (2) elderly patients and patients with good response to the first treatment were more likely to choose to continue BTX-A treatment, (3) multiple treatments of BTX-A remained effective within a group of TN patients who have chosen to continue BTX-A therapy, but efficacy decreased to a certain extent after multiple treatments.

After the first treatment, VAS decreased from 8 (6.75, 10) to 3 (2, 6.25) (*P* < 0.001). The degree of decrease in the score is sufficient to show the good analgesic effect of BTX-A. There was also a significant decrease in VAS in subgroups grouped by age. According to PGIC self-evaluation, 66.67% of the patients were improved after the first treatment. In our study, the longest duration of efficacy was 13 months.

The analgesic mechanisms of BTX-A proposed in existing studies include anti-inflammatory effects, inhibition of nociceptive neurotransmitter release, retrograde transport mechanism, suppression of ion channel activity, damaged nerve repair mechanisms, and modulation of central sensitization. The details are as follows. (1) Anti-inflammatory effects: Multiple studies have demonstrated that in rats with formalin-induced inflammatory pain models, injecting BTX-A into the plantar surface of the hindpaw produces antinociceptive effects lasting over 12 days. In a mouse model of TN, BTX-A injection into the vibrissal pad suppressed ipsilateral microglial activation and downregulated the upregulation of Toll-like receptor-2, myeloid differentiation factor downstream signaling, and pro-inflammatory cytokine expression (15, 16). (2) Inhibition of nociceptive neurotransmitter release: Nociceptive neurotransmitters play a critical role in pain signal transmission and perception. BTX-A inhibits the release of calcitonin gene-related peptide, glutamate, and Substance P (17). (3) Retrograde transport mechanism: Antonicci et al. (18) observed retrograde transport of BTX-A to the central nervous system via radiolabeling, showing that peripheral BTX-A exerts central effects through retrograde axonal transport and endocytosis. Wu et al. (19) demonstrated that subcutaneous BTX-A injection in the vibrissal pad of TN rats significantly elevated pain thresholds and enhanced antinociception. BTX-A is transported via axons to the caudal subnucleus of the trigeminal spinal nucleus, where it likely suppresses the overexpression of Transient Receptor Potential Ankyrin 1, Transient Receptor Potential Vanilloid 1, and Transient Receptor Potential Vanilloid 2, thereby reducing central sensitization. (4) Suppression of ion channel activity: In a rat model of TN, subcutaneous BTX-A injection significantly inhibited the expression of Voltage-gated Sodium Channel 1.7 in the trigeminal ganglia. This modulation reduces neuronal hyperexcitability and pain transmission (20). (5) Damaged nerve repair mechanisms: Schwann cells are pivotal in nerve repair, forming myelin sheaths, providing trophic support, and promoting axonal regeneration. In a mouse model of early nerve injury, BTX-A promoted Schwann cell proliferation and maturation (21). (6) Modulation of central sensitization: Endogenous opioid receptors play a central role in the modulation of pain perception, and it is an important component of the endogenous analgesic system. It was found that BTX-A was able to enhance the activity of endogenous opioid receptors (22).

Current clinical studies typically focus on the efficacy of single BTX-A treatments, with a maximum observation period of 14 months (14). But there were no studies on multiple treatments. The mean age of the TN-S group was significantly younger than the TN-M group, suggesting that older patients may be more likely to choose BTX-A treatment than younger patients. The safety and ease of BTX-A administration may also contribute to the preference for BTX-A therapy among older patients, and most diseases (e.g., hypertension, diabetes, liver or kidney dysfunction, or abnormal coagulation) are not contraindications to BTX-A treatment. According to our previous research (9), BTX-A is effective and safe for the treatment of patients of advanced age with ITN when used at dosages comparable to those used in younger patients, suggesting that BTX-A is likely to be a first-line treatment option for TN in older adults. Furthermore, the higher likelihood of elderly patients continuing treatment may partly be due to younger patients being more concerned about the potential aesthetic side effects.

Our study found that multiple BTX-A treatments remained effective, but efficacy decreased to a certain extent. Immunogenicity is a commonly encountered problem for the clinical use of many biological agents, and BTX-A is also immunogenic after prolonged use (23). Chronic exposure to botulinum toxin and the presence of stable neutralizing antibody titers may also contribute to the observed decline in efficacy (24). Various laboratory assays can be used to monitor antibody status in patients with possible immunoresistance. Attention should be paid to avoid large doses and repeated use in a short period of time. Poor or no response to BTX-A may also be due to improper BTX-A preparation or storage, insufficient dosing, inappropriate muscle selection, or improper injection techniques or targeting (23). These errors should be minimized during treatment, by additional training, better adherence to protocols, careful attention to patient responses and using electromyography.

In recent years, increasing evidences have shown that BTX-A has significant efficacy in the treatment of mental illness, especially depression (25). In previous studies on the treatment of TN with BTX-A, it was also found that BTX-A not only improved pain, but also significantly improved anxiety and depression (26). In this study, HAMA and HAMD scores of the two groups before and after treatment were also observed, but no significant difference was found. Firstly, the therapeutic injection for TN is administered at the pain site, whereas the injection site for anxiety and depression is multiple points in the frontotemporal region. This is based on the hypothesis of the “facial feedback” theory. The difference in injection sites leads to poor anti-anxiety and anti-depression effects. Secondly, this may be related to the low overall degree of anxiety and depression in patients before treatment. Some patients are not accompanied by anxiety and depression symptoms, so there is no improvement. Finally, a small sample size can result in insufficient statistical significance, making it possible that even if a true effect or relationship exists, it may not be detected. Therefore, it may be necessary to expand the sample size for further research and observation.

Nonetheless, our study has some limitations that should be considered. Firstly, due to its retrospective, unblinded, and uncontrolled design, the study lacks sufficient power to draw definitive conclusions. Hence, the results of our study need to be confirmed by placebo-controlled trials, and our sample size was relatively small, necessitating the use of a larger sample size in subsequent investigations. Secondly, because this is a retrospective study, selection bias may have occurred when patients chose to continue treatment. In addition, a proportion of patients were excluded due to a lack of follow-up data, which may also lead to selection bias.

## 5 Conclusion

In summary, BTX-A treatment was effective in TN. Elderly patients and patients with good response to the first treatment were more likely to choose to continue BTX-A multiple-treatments. BTX-A remains effective within a group of TN patients who have chosen to continue BTX-A therapy, but the efficacy decreases to a certain extent after multiple treatments. Since facial weakness is a complication of BTX-A treatment, large-scale studies including a comparison of two or three different doses are needed to identify the lowest effective dose. In the future, randomized, controlled trials of stratified dosing regimens and trials of immunogenicity monitoring are needed.

## Data Availability

The raw data supporting the conclusions of this article will be made available by the authors, without undue reservation.
